# Neurocognitive function in children with compensated hypothyroidism: lack of short term effects on or off thyroxin

**DOI:** 10.1186/1472-6823-6-2

**Published:** 2006-03-20

**Authors:** Naghma J Aijaz, Evelyn M Flaherty, Thomas Preston, Stacey Storch Bracken, Andrew H Lane, Thomas A Wilson

**Affiliations:** 1 Department of Pediatric Endocrinology, State University of New York, Stony Brook, NY, USA; 2 Department of Psychology, State University of New York, Stony Brook, NY, USA; 3 Department of Neurology, State University of New York, Stony Brook, NY and St. Charles Hospital, Port Jefferson, NY, USA

## Abstract

**Background:**

Although thyroxin therapy clearly is beneficial to children with frank hypothyroidism there is little data on the effects of thyroxin in children with compensated or subclinical hypothyroidism. The objective of this study was to determine the effect of thyroxin therapy on cognitive function in children with compensated hypothyroidism. The hypothesis was that thyroxin therapy would change neuropsychological function.

**Methods:**

Eleven patients with a history of sub clinical hypothyroidism entered the study. At the start of the study, six out of the 11 were on thyroxin therapy, while 5 were off therapy. All patients underwent a battery of neuropsychological testing and thyroid function tests at the start of study. Based on the results of thyroid function tests, two of the 5 patients who were off thyroxin were started back on thyroxin. All of the 6 patients who were on thyroxin were taken off thyroxin. All patients then underwent repeat neuropsychological testing and thyroid functions after an average of 91 days.

**Results:**

Thyroxin therapy could not be shown to have an effect on neuropsychological function in this short term study. Our patients had attention problems as compared to the normal population. No significant differences were found between our subjects and normal population standards in verbal processing, visual processing, motor speed/coordination and achievement.

**Conclusion:**

In this small, short term study, thyroxin therapy could not be shown to affect neuropsychological function in children with compensated hypothyroidism. These children may have attention problems but appear to have normal verbal and visual processing, motor speed/coordination and achievement.

## Background

A strong relationship exits between thyroid hormone and brain development. Thyroid hormone therapy has a significant long-term impact on child behavior, locomotor ability, speech, hearing and cognition in children with congenital hypothyroidism [1]. In addition, behavioral problems were a common finding in children and adults with congenital hypothyroidism before the era of neonatal screening [2,3]. While Ilicki and Larsson reported that Swedish children with congenital hypothyroidism who were diagnosed by neonatal screen and were given early treatment displayed average development as compared to the normal population [4], other studies have demonstrated persistent deficits. A recent long term follow up study of children with congenital hypothyroidism by Rovet suggested that while early detection and treatment improves intellectual functioning, non-verbal and visuo-spatial abilities are significantly below that of their siblings, and the differences increase with age [5]. Song et al. showed that neuropsychological function of children with congenital hypothyroidism in the post neonatal screening era was affected by factors associated with etiology of disease and timing of normalization of TSH by thyroxin treatment [6]. Bargagna et al. showed that 20% of children with congenital hypothyroidism detected by neonatal screen and treated early in life showed a generalized learning disorder [7]. Simons et al. concluded that children with severe congenital hypothyroidism treated early at mean age of 27.5 days (19–57 days) showed deficits in IQ score at age 3 and 5 year of age and these deficits were still evident at 10 years of age [8]. Derksen-Lubsen and Verkerk performed a meta-analysis of data based on seven studies which were carried on patients with congenital hypothyroidism detected by screening and treated early in life. The meta-analysis demonstrated IQ deficits despite early detection and treatment [9]. Kooistra et al. evaluated behavioral characteristics of children with congenital hypothyroidism and concluded that "early treated congenital hypothyroidism is associated with introversion rather than with social negativity" [10].

The studies mentioned above assessed the relationship between hypothyroidism and psychomotor development in children with overt congenital hypothyroidism characterized by clear elevations of serum TSH concentration and low serum T4 concentrations. Data on outcomes of children with more subtle forms of hypothyroidism are sparse. Compensated hypothyroidism is defined as a normal serum thyroxin concentration, a slightly elevated TSH, and absence of clinical features of hypothyroidism. Compensated hypothyroidism is sometimes referred to as "sub clinical hypothyroidism" or "hyperthyrotropinemia". Studies of compensated hypothyroidism in children are few and limited to the natural history [11]. To date, no study has examined neurocognitive function and the impact of thyroxin therapy in children with compensated hypothyroidism. This study evaluated cognitive function and the effect of thyroxin therapy on cognitive function in children with compensated hypothyroidism.

## Methods

### Subjects

Charts of 21 patients identified with compensated hypothyroidism seen in our institution between 1989 and 2002 were reviewed. Inclusion criteria for compensated congenital hypothyroidism were: subjects with a T4 concentration within the normal range and a TSH concentration greater than the laboratory normal range but less than 40 mcU/ml on confirmatory testing. Subjects with compensated acquired hypothyroidism were identified as children > 1 yr of age whose serum concentration of T-4 was in the normal range and TSH above the laboratory normal range but <20 mcU/ml. We excluded subjects with central hypothyroidism and those at risk for abnormal psychomotor development for reasons other than congenital hypothyroidism (i.e. chromosomal abnormalities, syndromes). Study protocol and consents were approved by the Stony Brook Committee on Research Involving Human Subjects. Parental consent was obtained for all subjects and assent was obtained for those over 12 years of age.

### Protocol

Subjects were divided into two groups: Group 1 was on thyroxin therapy at the start of the study and Group 2 was not on thyroxin therapy at the start of the study. All underwent neuropsychological testing and thyroid function testing at the beginning and the end of the study and each subject served as his/her own control. After initial testing, those in Group 1 were taken off thyroxin. Thyroid function tests were repeated in 6–8 weeks to check their thyroid status and neuropsychological testing was repeated. Thyroxin therapy was resumed if their TSH was elevated. Those in Group 2 also underwent thyroid function assessment and neuropsychological testing at baseline, after which they were started on thyroxin therapy if the TSH was still elevated. In 6–8 weeks, thyroid status was re-checked and neuropsychological testing repeated. The repetition of the neuropsychological testing was necessary to determine the effect of thyroxin therapy on neuropsychological function, and in those who remained off thyroxin therapy, to determine the practice effect that may occur with repeat neuropsychological testing. Scores on the various neuropsychological studies were also compared to age matched normative data derived from the general population.

### Hormone assays

Thyroid function tests were done by commercial assays as determined by subjects' insurance but were done in the same laboratory for each subject.

### Neuropsychological testing

Neuropsychological tests included the following:

1. Differential Abilities Scale (DAS) for general cognitive function

2. Test of Variables of Attention (TOVA), a measure of sustained attention

3. Tower of Hanoi (TOH), a measure of problem solving, mental flexibility and working memory

4. Controlled Oral Word Association (COWA), a test of verbal fluency

5. Purdue Pegboard is a measure of fine motor speed and dexterity

6. Child Behavior Checklists and Profile (The Teacher Report Form could not be given as most of the data collection took place in the summer months)

7. Woodcock-Johnson psycho educational battery- 3^rd ^edition (WJIII) to assess various aspects of scholastic achievement

8. Children's Memory Scale (CMS), a test of verbal and non-verbal learning and memory

9. Judgment of Line Orientation, a non-motor test of spatial judgment

### Statistical analysis

The data were analyzed using a repeated measures "Multivariate Analysis of Variance" (MANOVA) test. Chi-square analyses were conducted to determine whether the proportion of children in the current sample were similar or discrepant in functioning from that which would be expected in a normal population.

Because of the small sample size, the numbers of variables under investigation were reduced by the creation of composites. Table I indicates the composite variables and the variables from which they were formulated. The composites for each visit were calculated by the arithmetic means of the variables. Since the age range of children studied was considerable and not all children were able to perform all tests (e.g. Judgment of Line Orientation), some scores were not available. In these cases, the available scores were used to represent the mean overall performance on that composite. Because of the apparent randomness of the missing data, it is unlikely that such estimation techniques have skewed the findings.

**Table 1:** Composite Variables and Individual Subtests

Verbal Processing	
DAS Verbal	
CMS Verbal Memory	
Controlled oral word association: animals	
Visual Processing/Reasoning	
DAS Nonverbal Reasoning	
DAS Spatial	
CMS Visual Memory	
Attention/Executive Processing Efficiency	
TOVA	
Motor Speed/Coordination	
Purdue Pegboard	
Academic Achievement	
Reading – WJIII Letter Word Identification, Passage Comprehension, Word Attack	
Mathematics – WJIII Calculation, Applied Problems	
Spelling – WJIII	

## Results

Of 21 patients identified with compensated hypothyroidism, parents of 14 consented for the study. Eleven completed the study. Eight of the 11 were diagnosed with congenital compensated hypothyroidism (diagnosed at less than 3 months of age). All had been treated with thyroxin from infancy but four of these 8 had been taken off thyroxin therapy after age 3 yr. The other three had acquired compensated hypothyroidism (diagnosed age > 1 year of age). Two of these 3 were on thyroxin at entry into the study. BMI of the 11 subjects varied from the 22^nd ^to 97^th^% for age (mean ± SD = 64.5 ± 27.9). Thus, none were obese. Additional characteristics of the patients are summarized in Table II.

**Table 2:** Characteristics of subjects. Parentheses indicate range. To convert T4 to mcg/dl divide by 12.87

	#	Age at diagnosis	T-4 at diagnosis nmol/L	TSH at diagnosis mcu/ml	Age at initial Rx
Congenital	8	1.0 month (0.4–2.1)	117.1 (75.9–150.5)	11 (7.4–14.9)	1.3 months (0.5–2.6)
Acquired	3	6.8 years (1.2–12.3)	69.5 (65.6–73.4)	8.2 (5.4–12.1)	6.8 years (1.2–12.3)

At the start of the study, 6 patients were on thyroxin therapy (Group 1) and 5 patients were not on thyroxin therapy (Group 2). The mean age at the start of the study was 8.1 years (3.6–12.6). Group 1 had a mean T-4 of 128.7 nmol/L (10 mcg/dl) (range: 87.5 – 164.7 nmol/L [6.8–12.8 mcg/dl]) and mean TSH of 2.7 miu/ml (range 0.97–5.5) at the start of the study. Group 2 had a mean T-4 of 91.4 nmol/L (7.1 mcg/dl) (range 69.5–113.3 nmol/L [5.4–8.8 mcg/dl]) and mean TSH of 5 miu/ml (range 2.6–9.4) at the start of the study. One patient from group 2 declined blood workup prior to the neuropsychological testing. Of the 5 subjects in group 2, two were placed back on thyroxin after the first neuropsychological testing because of an elevated TSH. The remaining 3 now had normal thyroid function tests and therefore were not placed on thyroxin. The overall mean time period between the visit 1 and visit 2 for the neuropsychological tests was 91 days (60–131).

### Effect of thyroxin

Multivariate Analysis of Variance (MANOVA) was conducted to determine if there was a significant difference in performance on the major composite variables due to drug status (on/off thyroxin). A separate MANOVA was performed for each of the two time points. No significant differences were found due to being on or off thyroxin at either Visit 1 or 2 for any of the composite variables. Table III indicates the means for those children on and off thyroxin at each of the visits.

**Table 3:** Composite Means and SD (in parentheses) for Children On or Off Thyroxin

	Visit 1			Visit 2			
			
Major Area of Functioning	On (n = 6)	Off (n = 5)	All (n = 11)	On (n = 2)	Off (n = 9)	All (n = 11)	All (n = 11)
Verbal Processing	97.92 (12.97)	94.57 (9.22)	96.39 (11.01)	98.00 (10.84)	104.74 (9.31)	103.51 (9.41)	99.95 (8.95)
Visual Processing/Reasoning	100.28 (17.59)	100.85 (7.92)	100.54 (13.41)	104.17 (29.93)	102.82 (15.52)	103.06 (16.81)	101.80 (14.46)
Attention/Executive Processing Efficiency	77.69 (8.31)	80.52 (16.33)	79.26 (12.71)	80.90 (19.66)	80.63 (21.19)	80.68 (19.80)	81.77 (17.66)
Motor Speed/Coordination	93.07 (12.00)	94.00 (11.12)	93.53 (10.92)	96.00 (13.67)	85.70 (17.94)	87.58 (17.13)	89.47 (14.10)
Academic Achievement	109.90 (12.73)	100.07 (7.68)	105.43 (11.45)	99.50 (12.49)	105.04 (11.48)	104.03 (11.22)	104.73 (11.24)

Note: Means presented in standard scores, 100 = 50^th ^percentile. N's may vary.

A repeated measures analysis of variance was also conducted to determine if children performed differently at the two time points. Again, there were no significant differences for any of the major composite variables. Mean scores at time 1 were not significantly different from those at time 2.

Because some children were off thyroxin at both visits, a separate MANOVA was conducted to determine whether the scores of this group of children differed from the scores of children from the other two groups (those that had either stopped taking the drug after baseline or had started it after the baseline neuropsychological tests were completed). Results indicated that this group did not differ significantly from the other two groups.

Although our overall findings showed no effect of either drug or time in this sample, one non-significant trend was noted. The two children who were off thyroxin at baseline and were on thyroxin at the second study visit, showed a trend of higher visual processing scores at the second study visit. Means for visits one and two were 96.92 (SD = 9.78) and 104.17 (SD = 29.93), respectively.

### Cognitive and behavioral findings as compared to the normal distribution

Additional analyses were conducted to determine if this sample of children showed differences on the major composite variables as compared to what might be expected from a distribution of normal children. To analyze these data, scores on the five major composite variables were collapsed across visits one and two to create total composite scores (See Table III).

Chi-square analyses were conducted to determine whether the proportion of children in the current sample demonstrated similar or discrepant functioning from what would be expected in a normal distribution. A cut-off of 1.5 standard deviations below the mean was chosen to represent significantly poorer functioning. This cut off corresponds to a standard score of 78 and represents the lowest 13.59 % of children in a normal distribution. Several interesting findings from the chi-square analyses are presented below:

### Visual processing

Based on the mean composite scores, visual processing was not significantly different from verbal processing. This finding was surprising based on previous research indicating that children with congenital hypothyroidism showed relative deficits in nonverbal/visual processing skills. Data for Judgment of Line Orientation represented too few subjects (n = 6) to be included in the overall visual processing composite but were reviewed separately. The strikingly low mean for the Judgment of Line Orientation test (Mean = 73, SD = 23) indicated the possibility of problematic visual processing despite the absence of an overall deficit on the visual processing composite.

### Verbal processing

No significant difference was observed on the verbal processing composite as compared to the normal distribution. Results indicated overall average verbal abilities in this sample.

### Attention/Executive/Processing

#### TOVA

Results of the TOVA were most striking in comparison to the normal distribution based on TOVA normative data. The chi-square analysis showed that composite scores were well below that expected of children in a normal population, χ^2 ^= 84.02, df = 1, p < .01. More detailed investigation of this finding, through inspection of specific TOVA variables, is worthy of discussion. The TOVA contains five major variables: (1) Omission errors (Inattention); (2) Commission errors (Impulsivity); (3) Response Time; (4) Variability in Response Time; and (5) Perceptual Sensitivity. Our subjects demonstrated two major patterns of difficulty. First, across the whole of the test, they demonstrated abnormalities in Omissions, Response Speed, Variability and Perceptual Sensitivity, rather than in Commissions. On the other hand, a majority of the patients also committed an abnormal number of anticipatory responses during the second half of the test, which tends to draw such impulsivity by presenting the target stimulus more frequently in a "Go-No Go" paradigm. Thus they appear to have displayed a mix of deficient information processing and, when provoked, impulsivity.

#### Tower of hanoi visit 1

Data from the Tower of Hanoi test were analyzed separately because of concerns of practice effects from visit one to visit two. These indicated a significant chi-square of χ^2 ^= 15.31, df = 1, p < .01. Scores on Tower 1 were significantly lower than would be expected as compared to a normal distribution. This test involves complex attention and working memory, and the results thus once again suggest deficits among these children in these areas. It is worth noting further that our subjects tended to fare poorly on the Tower of Hanoi not because they made impulsive (i.e., rule-breaking) errors, but rather because they were unable to solve the problems efficiently.

### Motor Speed/Coordination

The motor speed /coordination composite scores did not indicate a significant difference as compared to the normal distribution.

### Achievement

Scores on the composite achievement did not differ as compared to scores expected from a normal distribution

### Behavioral functioning

Based on the CBCL, subject profiles were indicative of very slight elevations in the attention domain, but these were not significantly worse that the instrument's norms. In addition, no significant changes were seen on the CBCL across the course of the study.

## Discussion

As primary outcome measure, this study did not reveal an influence of thyroxin therapy on neurocognitive function in this small group of children with compensated hypothyroidism. Similar studies of the effect of thyroxin supplementation on neurocognitive function in adults with subclinical hypothyroidism have revealed variable results, but the largest study found no effect (studies summarized in ref. 12). The developing brain is known to be more sensitive to thyroxin deprivation than the adult brain, hence this remains an open question in children. Since this was a short term study, it is possible that a longer period of being on or off thyroxin therapy may have yielded different results. However, for safety considerations, it was considered important to carry out a short term study before examining the effects of thyroxin withdrawal and therapy over a longer period in this population.

When compared to normative data, the neurocognitive function testing suggest that even though they may not be classifiable as having ADHD, our subjects with a history of compensated hypothyroidism have attention problems. This is most evident on the TOVA, where a majority of our subjects performed abnormally, and to a lesser degree on the TOH, where a significant minority performed outside the normal range on the first administration. These attentional findings are best described as "sub clinical," in the sense that they were not confirmed by parent ratings of behavior in the home.

The specific abnormalities seen on the TOVA and Tower of Hanoi are worthy of comment. Findings on the TOVA were mixed, partially indicative of "inattention" and "slowed and variable processing", i.e., a large proportion of omission errors, and slow and variable reaction time; and partially indicative of "impulsivity" in the sense that a majority of these subjects made anticipatory errors during the second half of the test (which provokes these impulsive errors through frequent target presentation). Tower of Hanoi findings were mainly notable for inefficient problem solving rather than impulsive behavior.

There is currently considerable debate within the field of ADHD research as to the validity of different types of ADHD categories (Hyperactive, Inattentive, and Combined). Barkley have suggested that the "Inattentive Type" of ADHD may ultimately be reclassified as an independent disorder involving cognitive pacing and processing, while ADHD itself should be more narrowly described as a disorder fundamentally involving impulse control and self-regulation (13,14). While our sample size is too small to provide definite information regarding the exact type of attention/impulse control problems these children have, the data do suggest that their basic difficulties may have more to do with efficient information processing and problem solving than to impulse control and self-regulatory functions.

There is controversy concerning the relationship between thyroid dysfunction and ADHD. To date, the only clear association between thyroid dysfunction and ADHD is seen in children with the syndrome of thyroid hormone resistance [15]. A recent study found no correlation between T4 concentrations on neonatal screening and the later development of ADHD [16]. However, this study did not report TSH concentration, which is generally a more sensitive indicator of thyroid dysfunction, and therefore may have missed those with mild thyroid dysfunction. A study in rats has shown that transient thyroid depletion in the newborn period is associated with attention deficit like behavior later in life [17].

Two important limitations exist in our study. Our subjects' combined composite scores were compared to normative data derived from the general population rather than to a control group obtained within the study. In addition, it is possible that those parents who agreed to enter their children in the study had doubts about neurodevelopment of their off spring thus introducing a selection bias. However, this scenario was not suggested by parent's behavior rating at home. The children with congenital compensated hypothyroidism were ascertained before 2000. Since that time, TSH norms for infants have been broadened in many laboratories (for example: ). Thus by current laboratory standards, some of the infants we diagnosed with compensated hypothyroidism prior to 2000 would now be considered normal. However, 5 of the 8 children with compensated congenital hypothyroidism in our study had been tried off Thyroxin therapy at a mean age of 3.5 years. Their l-T4 was 97.8 nmol/L (7.6 mcg/dl) (range 82.4–122.3 nmol/L [6.4–9.5 mcg/dl]) and TSH was 8.8 mcU/ml (range 4.8–12). Three of the 5 had been restarted on thyroxin because of the TSH was still elevated suggesting that mild elevations of TSH in infancy may signal persistent elevations in TSH later in childhood. The fact that we found significant deficits in attention and problem solving suggests the possibility that even minor elevations in TSH may be significant.

## Conclusion

This study suggests that, despite the absence of behavioral manifestations of distractibility and hyperactivity, children with a history of compensated hypothyroidism may have significant deficits in attention on clinical measures (TOVA, TOH) relative to the normal distribution as defined. However, over the short term, thyroxin replacement does not seem to have an impact on neuropsychological function.

## Abbreviations

T4: Thyroxin

TOH: Tower of Hanoi

TOVA: Test of Variables of Attention

TSH: Thyroid stimulating hormone

## Competing interests

The author(s) declare that they have no competing interests.

## Authors' contributions

Naghma Aijaz, Thomas Preston and Thomas Wilson conceptualized and planned the study.

Naghma Aijaz, Andrew Lane and Thomas Wilson identified and recruited subjects for the study and provided medical care to them during the study.

Stacey Bracken and Evelyn Flaherty carried out the battery of psychological tests.

Evelyn Flaherty, Naghma Aijaz and Thomas Preston performed the statistical analysis.

All authors contributed to writing the manuscript.

## Funding

This study was not funded by any agency or organization. No agency or organization had any role in the design, accomplishment, or interpretation of the study or in the preparation of the manuscript.

## Pre-publication history

The pre-publication history for this paper can be accessed here:


